# Cytomegalovirus in Ulcerative Colitis: An Unwanted “Guest”

**DOI:** 10.3390/pathogens13080650

**Published:** 2024-08-02

**Authors:** Danusia Onisor, Olga Brusnic, Simona Mocan, Mircea Stoian, Calin Avram, Adrian Boicean, Daniela Dobru

**Affiliations:** 1 Department of Internal Medicine VII, George Emil Palade University of Medicine, Pharmacy, Science and Technology of Targu Mures, Gheorghe Marinescu Street No. 38, 540139 Targu Mures, Romania; danusia.onisor@umfst.ro (D.O.); daniela.dobru@umfst.ro (D.D.); 2 Gastroenterology Department, Mureș County Clinical Hospital, 540103 Targu Mures, Romania; 3 Pathology Department, Emergency County Hospital, 540136 Targu Mures, Romania; slmocan@yahoo.com; 4 Department of Anesthesiology and Intensive Care, George Emil Palade University of Medicine, Pharmacy, Science and Technology of Targu Mures, 540139 Targu Mures, Romania; mircea.stoian@umfst.ro; 5 Department of Medical Informatics and Biostatistics, George Emil Palade University of Medicine, Pharmacy, Science and Technology of Targu Mures, Gheorghe Marinescu Street No. 38, 540139 Targu Mures, Romania; 6 Faculty of Medicine, Lucian Blaga University of Sibiu, 550169 Sibiu, Romania; adrian.boicean@ulbsibiu.ro

**Keywords:** CMV, ulcerative colitis, diagnostic, treatment

## Abstract

The role of cytomegalovirus (CMV) in the flare-up of ulcerative colitis (UC) is not clearly understood. CMV can cause similar symptoms in different clinical contexts, which may be attributed to the natural evolution of the viral infection, the patient’s immune status, or its association with inflammatory bowel disease (IBD). This study aims to delineate the diverse manifestations of CMV-related lesions from clinical, endoscopic, and histopathological perspectives, alongside a brief narrative review of the literature. In managing IBD patients, it is crucial to be vigilant for signs of CMV reactivation, especially before the initiation of more intensive therapies.

## 1. Background

Cytomegalovirus (CMV) is a ubiquitous double-stranded DNA virus [1], an opportunistic pathogen, and a member of the herpesvirus family—specifically, the beta *Herpesviridae* sub-family [2]. It is a species-specific host-virus and can be found in various mammals, such as humans (HCMV), mice (MCMV), primates, and rats [3]. Over time, CMV has co-evolved with its host organism, and if it crosses over to another species in vivo, it cannot fully replicate [4]. The global prevalence of HCMV exposure in the population varies between 40 and 100%, depending on the level of industrialization and development in a specific region [5], with prevalence increasing with age [6]. Primary HCMV infection in immunocompetent adults is often asymptomatic [7] or may cause mild symptoms resembling mononucleosis [6,8], occasionally presenting systemic symptoms like fever, leucopenia, and splenomegaly [9]. In pregnant women, it can result in severe symptomatic congenital infections in the fetus and newborn [7]. Following the primary infection, in the majority of cases, HCMV remains in the body for the whole life in a latent state [10]. HCMV is known to persist latently in various cell types, including myeloid cells (specifically, hematopoietic stem cells), fibroblasts [11], and even colonic mucosa cells [6]. However, gaps in our understanding of HCMV latency exist, as it remains unclear how the virus persists in nondividing cells or evades cell division through unknown mechanisms [11]. While HCMV reactivation is very rare in immunocompetent patients [7], it can occur more frequently under conditions of compromised immune responses (such as AIDS, corticosteroid therapy, or treatment with immunosuppressants). The reactivation of HCMV can lead to a systemic disease characterized by fever, pancytopenia, and inflammatory changes in multiple organs [12], notably affecting the liver, lungs, retina [8], and, most commonly, the gastrointestinal tract [13]. Colitis is a common manifestation of this systemic disease [14,15]. In the literature, there are still ongoing debates about the influence of HCMV on the development of inflammatory bowel disease [16]. Some authors suggest that HCMV actively contributes to inflammatory flares, while others argue for the concept of HCMV as an innocent bystander not having any role in the progression of the disease just by being reactivated due to local inflammation [16,17,18]. If HCMV does play an active role, researchers are investigating the specific viral load that might trigger a flare in ulcerative colitis (UC) patients [19] as well as when treatment is necessary versus when spontaneous resolution can occur [20]. More commonly, HCMV reactivation in patients with UC is believed to be prompted by the upregulation of inflammatory mediators, leading to a vicious cycle where inflammation triggers the production of cytokines like interleukin-1 beta, interleukin-6, and, particularly, TNF alpha. These cytokines support viral expression and replication, exacerbating colon inflammation [14,19]. Research using a murine model by Nakase and colleagues indicated that MCMV infects specifically inflamed cells, suggesting that the spread of HCMV infection in UC could extend from perivascular stromal cells to endothelial and epithelial cells [2]. The risk of HCMV reactivation is higher in UC patients compared to in those with Crohn’s disease, particularly in patients with severe UC as opposed mild to moderate cases. Studies have shown that the risk of CMV reactivation is significantly elevated (2.108 times higher) [21] in patients with pancolitis compared to those with left-colon lesions, and the use of glucocorticoid therapy further increases this risk (4.175 times higher) [21]. While not all studies are in agreement, most of them indicate a higher mortality rate in patients with inflammatory bowel disease (IBD) [22,23], as well as increased resistance to treatment [6,20]. Treatment for HCMV has been shown to improve the prognosis [14,23], restore responsiveness to corticosteroid therapy, and reduce the need for colectomies [24,25]. Therefore, the early and accurate diagnosis of HCMV infections is vital in managing these patients.

The aim of this study is to describe patients with ulcerative colitis associated with HCMV infection and to provide a short narrative review of the literature.

## 2. Case Reports

### 2.1. Case 1

A 45-year-old woman with no significant medical history presented emergently to the hospital with symptoms of bloody diarrhea (8–10 stools/day), nausea, abdominal pain in the lower left quadrant, and a substantial weight loss of 15 kg over three months, with a BMI of 20. A physical examination revealed dehydration, abdominal pain and tenderness, and diminished bowel sounds. Paraclinical examinations highlighted hypochromic microcytic anemia, leukocytosis with neutrophilia, severe dyselectrolytemia, and hypoproteinemia with hypoalbuminemia. The fecal calprotectin was 3800 µg/g. The stool cultures for bacteria were negative. Ultrasonography revealed the thickening of the colonic wall, the loss of haustration, and a hypoechoic appearance, highly suggestive of ulcerative colitis. Due to a low performance status, rectosigmoidoscopy was chosen, and bowel preparation was performed using only enemas. The endoscopic appearance was indicative of ulcerative colitis, with granular, friable mucosa devoid of a vascular pattern. Three biopsies were taken.

**Figure 1:**
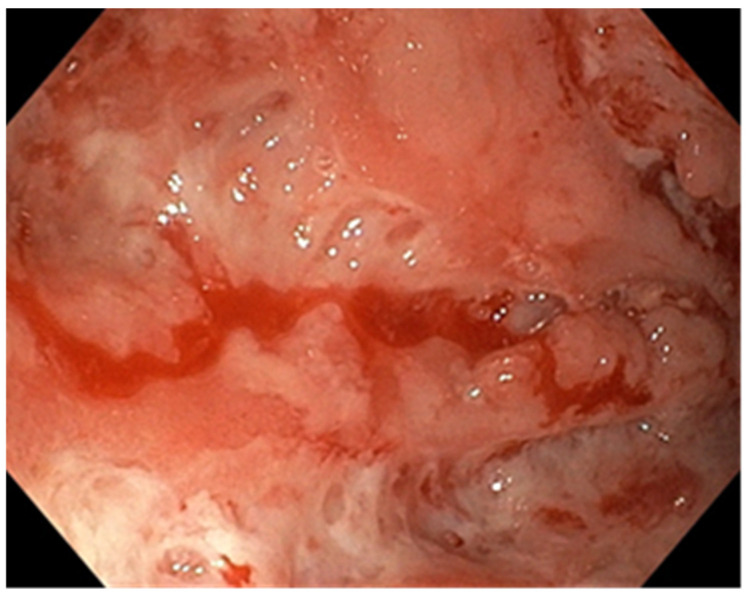
Endoscopic aspects—longitudinal and deep ulcers.

**Figure 2:**
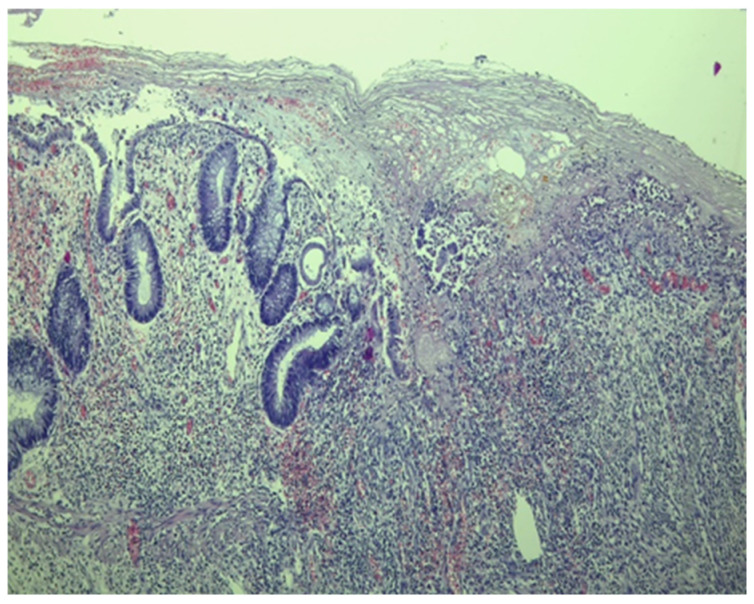
Fragment of colonic mucosa with marked architectural distortion associated with abundant chronic inflammatory infiltrate, including basal plasmacytosis and areas of ulceration (Hematoxylin eosin staining, ×4).

**Figure 3:**
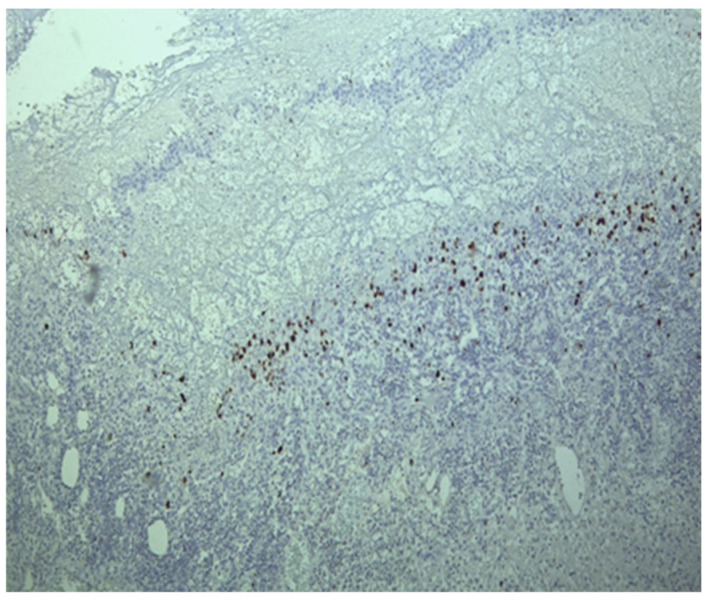
In the area of ulceration, numerous viral inclusions were detected by immunostaining.

Histological examination revealed crypt architectural distortion, associated with extensive chronic inflammation, including basal plasmacytosis, and active inflammation in the form of cryptitis and crypt abscesses, thus confirming the clinical diagnosis of ulcerative colitis. Treatment was initiated with corticosteroids, 5-ASA, and supportive therapy. Due to partial symptomatic improvement, the patient was discharged with treatment recommendations and follow-up instructions. One month later, she was readmitted due to the recurrence of symptoms. Ileocolonoscopy revealed persistent pancolitis with severe endoscopic activity characterized by longitudinal and deep ulcers (Mayo score of 10 points), as shown in Figure 1. Nine serial biopsies were taken: two from the ascending colon, two from the transverse colon, and five from the left colon, including from the bottom of the ulcers. Microscopically, the same pattern of chronic inflammation and associated ulcerations was observed. Notably, within the endothelial cells of the granulation tissue from the ulcerations, a high number of viral inclusions were identified, which tested positive on specific immunostaining for HCMV (Figure 2 and Figure 3).

Treatment was initiated with Ganciclovir at a dose of 5 mg/kg every 12 h for 3 days, followed by Valganciclovir at a dose of 900 mg twice daily for 21 days, and continued with Mesalazine at a dose of 4 g/day, and tapered corticosteroid therapy and anti-TNF therapy with Infliximab 5 mg/kg were introduced. After one year of treatment, the patient showed no signs of recurrent infection and was in both clinical and endoscopic remission. The one-year follow-up colonoscopy revealed numerous pseudopolyps of varying sizes.

### 2.2. Case 2

**Figure 4:**
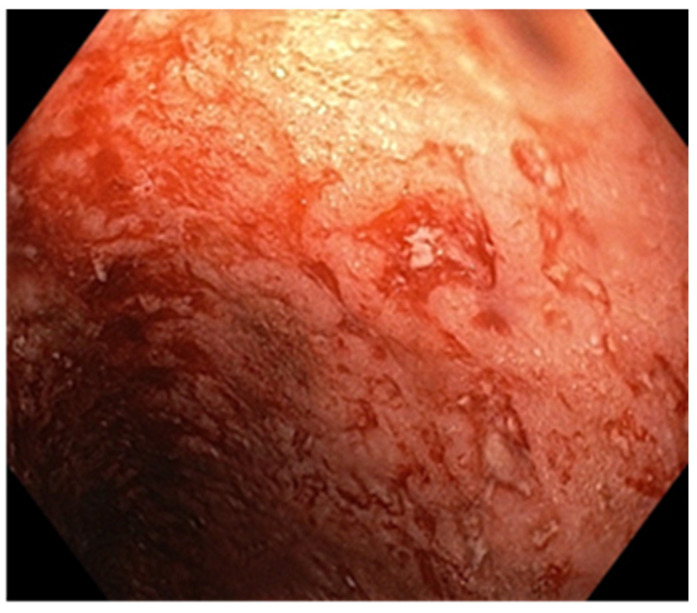
Endoscopic aspect—small and deep ulcers.

**Figure 5:**
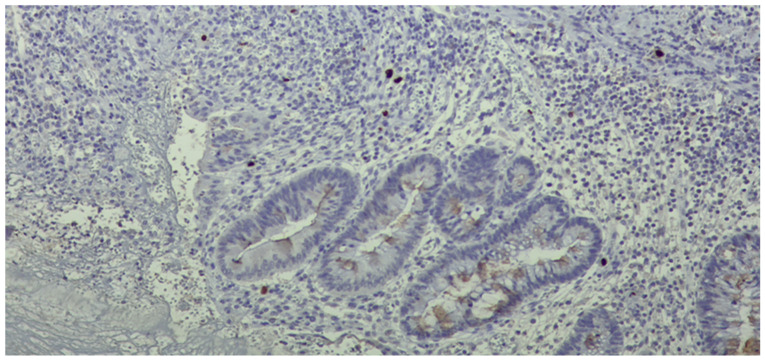
Immunostaining detected more than five viral inclusions in each fragment.

**Figure 6:**
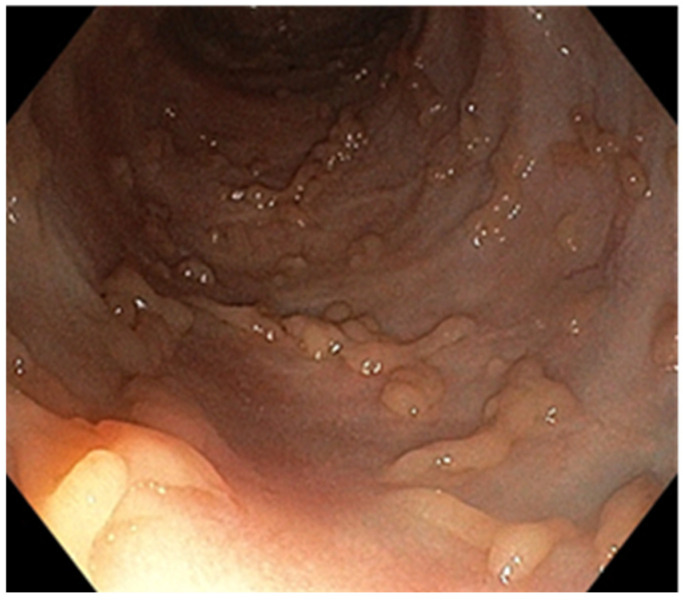
Endoscopic aspects at a one-year follow-up—pseudopolyps.

A 25-year-old man with a history of recurrent chronic pancolitis and backwash ileitis with one episode of exacerbation in the past three months, on a combined treatment of Adalimumab at a dose of 40 mg every two weeks, Azathioprine at a dose of 100 mg/day, and Mesalazine at a dose of 3 g/day, presented with diarrheal stools (seven to eight per day) containing mucus and blood, including nocturnal episodes, and weight loss (BMI 19). Laboratory tests revealed normochromic and normocytic anemia, thrombocytosis, dyselectrolotemia, hypoproteinemia with hypoalbuminemia, elevated ferritin levels, and a very high CRP. The fecal calprotectin was measured at 5400 µg/g. Anti-adalimumab antibodies were detected with a value of 7 (<10 AU/mL) and an Adalimumab trough level of 15 µg/mL. To determine the etiology of the current exacerbation, we performed stool bacterial and mycological examinations, as well as serology to identify any bacterial or viral infection. An increase in the HCMV IgG antibody titer to 480 AU/mL from an initial value of 190 AU/mL was observed, although HCMV IgM was negative. Given the high suspicion of superinfection with CMV, we performed an HCMV DNA test from the blood and obtained a result of 2820 IU/mL. For confirmation, we performed an ileocolonoscopy, which revealed continuous lesions, extending from the rectum to the terminal ileum. These lesions were characterized by erosions, small but deep ulcers, marked mucosal friability, and a lack of a vascular pattern, corresponding to Mayo 10p (Figure 4). Multiple biopsies were taken during the procedure, including from the edges and bottom of the ulcers. The histopathological examination confirmed our suspicion of diffuse chronic inflammation, with architectural distortions. Notably, we observed marked activity with crypt abscesses and ulcerations with granulation tissue formation. More than five viral inclusions were identified in the endothelial cells on multiple fragments, which tested positive for HCMV immunostaining (Figure 5). Due to the pandemic situation and the patient’s stable condition, we decided to discharge the patient and initiate treatment with Valganciclovir at a dose of 900 mg/day for three weeks. Azathioprine was discontinued, and we continued with Mesalazine at a dose of 4 g/day and Adalimumab at a dose of 40 mg every two weeks. The patient’s evolution improved. At the end of the therapy, HCMV-DNA was negative. Currently, the patient has no digestive problems and has gained weight. At a one-year follow-up colonoscopy, the mucosa showed multiple pseudopolyps of varying sizes, with no signs of infection relapse (Figure 6).

### 2.3. Case 3

A 41-year-old man with chronic recurrent ulcerative colitis (UC) on biological treatment with Adalimumab at a dose of 40 mg every 2 weeks and Mesalazine at a dose of 3 g/day presented with an exacerbation of symptoms, including watery diarrhea with minimal blood streaks, seven to eight times per day (including two nocturnal stools), abdominal pain in the left iliac fossa and right hypochondrium, fever (38.2 °C), and malaise. These symptoms began approximately 3 weeks ago.

**Figure 7:**
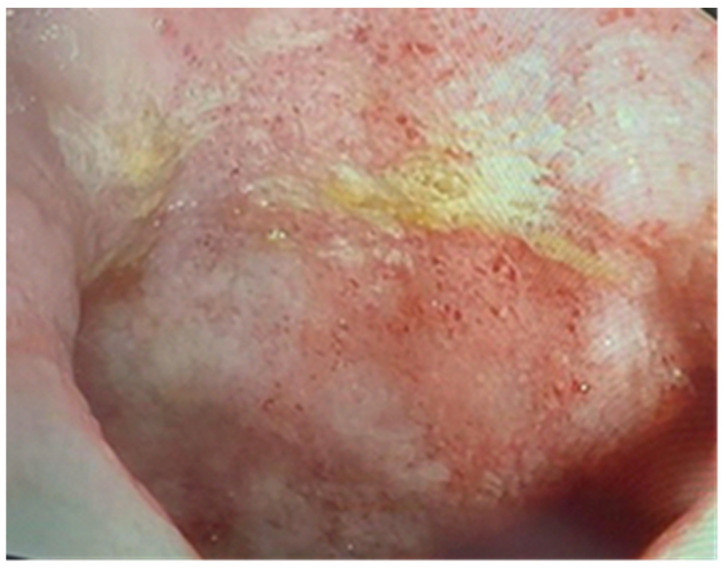
Endoscopic findings include erosions and a loss of the vascular pattern (atypical for HCMV colitis).

**Figure 8:**
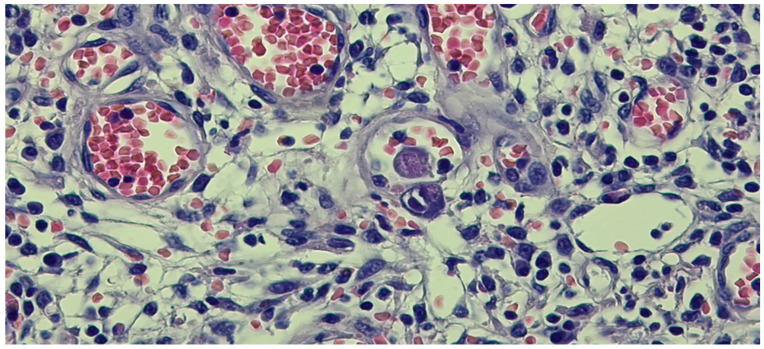
We identified isolated endothelial cells with viral inclusions in the granulation tissue (Hematoxylin eosin staining, ×40).

**Figure 9:**
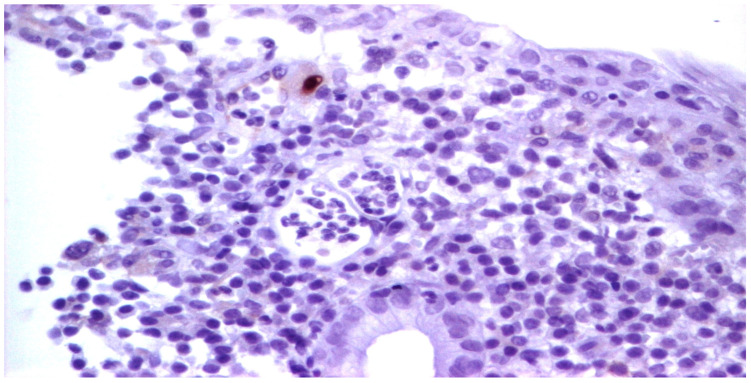
HCMV immunostaining pointing out rare viral inclusions in the endothelial cells.

Upon admission, laboratory tests revealed leukopenia, mild hypochromic normocytic anemia, thrombocytopenia, and signs of inflammation, cholestasis, and hepatocellular injury hepatic cytolysis syndrome. To determine the cause of the acute episode, we performed bacterial and mycological stool tests, as well as serological viral marker testing. We found an increase in HCMV IgG levels compared to the value at the initiation of biological therapy: 148 AU/mL and, later, 500 AU/mL. The plasma HCMV DNA was 7320 UI/mL. The HBs Antigen and HCV Antibody tests were negative. The value of anti-adalimumab antibodies was 0.5 (<10 AU/mL). Due to the biochemical changes in the liver, sclerosing cholangitis was initially suspected. However, this was ruled out with the aid of a cholangioMRI and negative pANCA values. Given these findings, HCMV hepatitis was suspected, and an ileocolonoscopy was performed. The endoscopic appearance was not characteristic of HCMV colitis, as there were erosions, ulcerations, a disappearance of the vascular pattern, and mucosal friability, the aspect being circumferential up to the level of the terminal ileum, corresponding to Mayo 8 points (Figure 7). Serial biopsies were collected, especially from the left side. Histopathological examination revealed a destructive pattern with chronic architectural changes and abundant chronic inflammation, including basal plasmocytosis and marked activity with crypt abscesses observed in all biopsy fragments. Rare HCVM viral inclusions were identified on HE staining at the level of the endothelial cells in the capillaries. The presence of the viral inclusions was confirmed by immunohistochemistry (Figure 8 and Figure 9). During hospitalization, the patient was treated with electrolyte solutions, and systemic therapy with intravenous ganciclovir was initiated at 5 mg/kg every 12 h for 3 days, followed by oral valganciglovir at a dose of 900 mg twice a day for 21 days. Additionally, the patient received Mesalazine at a dose of 3 g/day, and hepatoprotective measures were also continued, along with Adalimumab.

The patient showed clinical improvement with a reduction in the number of diarrheal stools, as well as biological improvement, as evidenced by relief from inflammatory symptoms and the normalization of liver samples. At the one-year follow-up colonoscopy, there were no signs of infection relapse, and the patient remained in clinical and endoscopic remission.

For the comfort of the patients, the colonoscopies were performed with propofol sedation, with careful monitoring of an anesthetist.

Ethics statement: The study was approved by the Ethics Committees of the County Clinical Hospital, Târgu Mureș (No. 4872/24.05.2022), and of the George Emil Palade University of Medicine, Pharmacy, Science and Technology, Târgu Mureș (No. 1804/22.06.2022).

All subjects provided informed consent.

## 3. Materials and Methods

We conducted a comprehensive search on the Web of Science, Scopus, and PubMed databases for articles published up to 1 January 2024 that examined the relationship between Cytomegalovirus and Ulcerative Colitis. The search terms used were “Cytomegalovirus” OR Cytomegaloviru colitis AND “Ulcerative colitis”. Our inclusion criteria comprised population studies involving adult humans, literature published in English, and research articles analyzing the correlation between HCMV and ulcerative colitis, including aspects of diagnosis and therapeutic management. We included full-length papers, retrospective cross-sectional studies, longitudinal studies, review articles, meta-analyses, and case reports. We excluded studies that did not align with the objectives of our article and non-English publications. To streamline the selection process, we utilized the Rayyan application. Each author reviewed the titles and abstracts of the retrieved records to eliminate irrelevant articles, and duplicates or triplicates were subsequently removed.

Our requirements were met by 49 articles.

Endoscopies were performed using an Olympus EVIS EXERA III videocolonoscope CF-HQ190L/I (OLYMPUS AMERICA INC., 3500 Corporate Parkway, PO Box 610, Center Valley, PA 18034, USA) and a Nikon Eclipse E800 (NIKON INSTRUMENTS INC. 1300 Walt Whitman Road, Melville, NY 11747-3064, USA) microscope was used for histopathological examinations.

## 4. Discussion and Narrative Literature Review

Detection of HCMV in flare-ups of UC can raise serious diagnostic and treatment problems as highlighted in the studies listed in Table 1 and which will be detailed later in this review.

HCMV infection plays a significant role in the evolution and treatment of patients with inflammatory bowel disease (IBD). This is because IBD patients are prone to HCMV reactivation due to inflammation and immunosuppression secondary to their treatment [7]. The main markers for evaluating inflammation are C-reactive protein, the erythrocyte sedimentation rate, fecal calprotectin, and fecal lactoferrin [25]. Besides the reactivation of a persistent virus, typically found in monocytes and endothelial cells, new strains can be introduced through blood transfusions or tissue transplantation [39]. UC, HCMV-colitis, and UC-HCMV colitis can present the same symptoms [6] and endoscopic appearance [6,35] but require different treatment.

### 4.1. Why UC?

Inflammation of the intestinal mucosa triggers the production of cytokines, including tumor necrosis factor (TNF-alpha), which stimulates viral replication and facilitates the migration of infected monocytes and macrophages into the inflamed tissue (where HCMV remains dormant). Despite similar rates of HCMV seroprevalence in both forms of IBD [40], Crohn’s disease (CD) and ulcerative colitis (UC), HCMV reactivation is significantly less common in CD compared to UC. All three patients in our study presented with ulcerative colitis, and we have not encountered any cases of Crohn’s disease (CD) with concurrent HCMV infection in our clinic. In a 7-year retrospective analysis conducted by Bonta et al., involving 14 patients with inflammatory bowel disease (IBD), only 2 were diagnosed with CD [20]. Additionally, McCurdy et al. observed, in a retrospective case-control study including 1111 patients, that 68 of them had HCMV infection, out of which 45 were diagnosed with UC and 21 were diagnosed with CD (*p* = 0.003) [31]. This difference is attributed to the distinct cytokine profiles involved in the respective inflammatory processes. In Crohn’s disease, there is an expression of Th1 and Th17 CD4+ helper T lymphocytes, which secrete interferon-gamma, playing a role in the inhibition of HCMV replication [17]. Conversely, in ulcerative colitis, there is an expression of Th2 lymphocytes, leading to the reduced secretion of antiviral cytokines and promoting viral reactivation and tolerance [41].

### 4.2. Onset and Risk Factors

In the case of the first patient, although the initial symptoms showed improvement, her condition deteriorated after a month. Following nine biopsies, the presence of HCMV infection was confirmed. The literature reports cases of the simultaneous onset of colitis with HCMV colitis occurring in approximately 4.5% of newly diagnosed UC cases [28,38,42]. Around 10% of UC patients develop HCMV colitis over time, with the incidence rising to 40% in cases of corticosteroid-resistant UC [22,38].

The heightened risk of HCMV reactivation is influenced by host factors, UC severity, and the treatment regimen. Host-related risk factors include: an age over 30 years [17,23,31] (OR 14.29; *p* = 0.004) [30], a disease duration of less than 60 months (OR 7.69; *p* = 0.011) [30], severity [21,33], and extension of the disease [10,21]. Qin et al. demonstrated that the risk of HCMV reactivation is 1.465 times higher in severe UC compared to that in mild and moderate forms and 2.108 times higher in patients with pancolitis compared to that in those with left-sided colitis [21]. Corticosteroid therapy is closely linked to HCMV activation, with a 4.17 times higher associated risk [21], which could be potentially linked to the dosage [23] and duration of the treatment [38]. Furthermore, HCMV activation can lead to the development of corticosteroid resistance through a mechanism that involves the alteration of glucocorticoid binding receptors from anti-inflammatory alpha to non-anti-inflammatory beta receptors [43].

Similarly, thiopurines (azathioprine, cyclosporine) increase the risk of HCMV reactivation because they disrupt the function of natural killer T lymphocytes, which are involved in the pathogenesis of HCMV reactivation [6,44]. Other studies, however, did not show a significant association between the two thiopurines [10]. Regarding the relationship between anti-TNF therapy and CMV reactivation, more studies found no causal effect [14,26], and Pillet et al. suggested that this medication could be used during flare-ups due to CMV reactivation [17]. Unlike anti-TNF, anti-integrin medication (vedolizumab) can lead to severe forms of HCMV reactivation [37,45]. Regarding the Janus kinase (JAK) inhibitor (Tofacitinib) and anti-interleukin 12/23 (Ustekinumab’s) study, there are not enough data to process.

### 4.3. Serology

Unfortunately, optimal diagnostic tests for CMV infection in the context of UC onset or relapse remain unclear. Two types of antibodies can be identified in peripheral blood by using the enzyme-linked immunosorbent assay (ELISA) technique. Specific IgG-CMV indicates patients who have been in contact with CMV and who could be at risk for reactivation of the infection [14]. On the other hand, IgM-HCMV is the marker not only for an acute primary infection but also for reactivation or reinfection, and it can persist in the peripheral blood for several months. However, the effectiveness of the examination can be affected by false positive reactions [17]. When IgM-HCMV is detected, it has a specificity and sensitivity of 100% and 99%, respectively, for acute systemic infection, but it lacks organ specificity. We did not detect positive IgM-HCMV in any cases, but in patients 2 and 3, we observed a doubling of the IgG-HCMV value, which raised the suspicion of HCMV reactivation. A review published by Yokoyama et al. in 2020 emphasized that IgG-HCMV shows small variations due to viral reactivation [36]. On the other hand, Nakase et al. highlight that a four-fold increase in the IgG-HCMV titer, 2–4 weeks apart, can be an indicator of viral reactivation [2]. For the last two patients, we performed HCMV-DNA testing from blood samples, resulting in values of 2820 IU/mL and 7320 IU/mL for the third patient. Unfortunately, there is no established cut-off level for blood HCMV DNA to distinguish latent from active infection [44]. Essen et al., in a study on 81 patients, reported a sensitivity of 66.7% and a specificity of 100% for values of ≥578 c/mL (895 IU/mL) [38]. Conversely, cut-offs in post-transplant patients vary from 4000 to 10.000 IU/mL [26]. The determination of the HCMV pp65 antigen can be conducted from serum or cerebrospinal fluid and has a sensitivity ranging from 60% to 100% and a specificity between 83% and 100% [46]. However, it is considered that the determination of DNA-HCMV and HCMV pp65 antigens in the blood does not always reflect the colon involvement [38]. Given the lack of uniformly established values for serological viral loads, an alternative technique was needed to confirm the mixed etiology of colitis.

### 4.4. Endoscopy and Biopsies Prelevation

The endoscopic appearance of UC-HCMV colitis can range from deep, well-defined, longitudinal ulcers (as seen in the first patient) to small ulcers (as observed in the second patient) and a granular, friable appearance with erosions (as seen in the third patient). These aspects can be challenging to differentiate from the appearance of active ulcerative colitis [6]. In the literature, the characteristics of endoscopic lesions are not specific [10] and can range from diffuse erythema with superficial erosions, to associated pseudomembranes, [10,47], to well-defined, longitudinal ulcers and a cobblestone-like appearance with a high sensitivity for HCMV presence which vary from 81 to 95% [47]. The presence of ulcerations with a distinct punched-out appearance is strongly linked to HCMV colitis (OR = 3.39, 95%, CI: 1.78–7.46), while a wide mucosal defect carries a 4.58-fold risk (95%, CI: 1.14–4.28) of being associated with HCMV colitis [10,47]. Similarly, a study conducted by Omiya et al. involving 20 patients suggested that the absence of large ulcers during endoscopic examination might indicate latent infection in patients with a positive mucosal viral test, rendering antiviral treatment ineffective [48]. On the other hand, Iida et al. pointed out that punched-out type ulcers are similarly detected in both HCMV-positive and HCMV-negative patients [49]. Additionally, there are conflicting results among studies that evaluated the severity of identified lesions and the presence of HCMV. Wada et al. found correlations between the severity of endoscopic lesions and the presence of HCMV, whereas Roblin et al. did not [27,50].

While endoscopic characteristics may raise suspicion, a definitive diagnosis requires histopathological evaluation. McCurdy et al., in a retrospective study that included 31 patients with UC, demonstrated that 11 biopsies are needed from the entire colon to achieve an 80% probability of at least one positive biopsy [31]. For patients with ulcerative colitis, European Crohn’s and Colitis Organization (ECCO) guidelines recommend flexible sigmoidoscopy, as studies show that viral antigens are not evident in biopsies from the right colon [44]. When ulcers are present, biopsy material must be collected from the depth or the edge of the ulcer [50]. The presence of HCMV is associated with inflammation [40], which is often focal [51], especially in treated patients. Sometimes, it is difficult to identify these focal points of inflammation endoscopically [17,19]. This difficulty is demonstrated in patients who undergo colectomy, where the virus is identified in the operative specimen but not in the pre-surgical biopsy [31].

### 4.5. Histhological Examination

Histopathological examination can reveal CMV infection reactivation by demonstrating the presence of viral antigens. In seropositive patients, there are two methods for determining infection reactivation in the colon by identifying the viral antigens [27]. Both methods rely on the biopsy material obtained during the colonoscopy. The first method involves the histopathological examination of the tissue. This detection can be carried out on standard Hematoxylin and Eosin-stained sections with a high specificity of 92–100% and a sensitivity between 10% and 87% [16] or through immunostaining, which can highlight the presence of modified cells containing viral inclusions, displaying the characteristic “owl eye” appearance with a good specificity of 92–100% and a sensitivity of 78–93% [52]. The second method involves detecting viral DNA in the colonic mucosa using in situ hybridization techniques or molecular determinations based on nucleic acid amplification tests [17]. The threshold value for a diagnosis is not yet well defined and ranges from 250 copies/mg [40] to 316 copies/mg [34] and 1000 copies/100,000 cells [29]. Consequently, patients are categorized into high-grade HCMV (tissue DNA ≥ 250/copies/mg or more than four inclusions on IHC) and low-grade HCMV (tissue DNA ≤ 250 copies/mg or less than four inclusions on IHC) [17,40]. The accuracy of identifying viral antigens depends primarily on the quality and quantity of the biopsy material examined [53]. To avoid false negative results, it is important to consider not only the number of fragments examined but also the specific location where they were collected and the time of collection in relation to the disease progression [18]. Nguyen et al. distinguished low-grade HCMV infection (positive only by IHC) from high-grade infection (detected by HE staining) [18]. On the other hand, Jones et al. and Kuwabara et al. defined increased HCMV density as the presence of 4 or more than 10 inclusions in the histological section [32,54]. The lack of a standardized classification system makes it challenging to develop the most accurate guidelines. In the case of the three patients discussed before, characteristic HCMV lesions were found in the left colon.

### 4.6. Treatment

According to the European Crohn’s and Colitis Organisation guidelines, the treatment of choice for UC-HCMV colitis is intravenous ganciclovir at a dose of 5 mg/kg twice daily for 5–10 days, followed by valganciclovir at a dose of 900 mg daily until completing a 2–3-week course [44]. In a randomized trial, Asberg et al. demonstrated that oral valganciclovir has a similar efficacy to intravenously administered ganciclovir in organ transplant patients [55]. Common side effects of ganciclovir, such as neutropenia and thrombocytopenia, are similar to the systemic effects of HCMV and can complicate management [44]. Prior to using ganciclovir for the treatment of UC-HCMV colitis, surgical intervention was necessary in 80% of patients, and the mortality rate was up to 33% [56] On the other hand, several studies have found no significant effect regarding antiviral treatment on the colectomy rate in UC-HCMV colitis with low-grade disease [18,32], but in patients with high-grade disease, the rates were 44% versus 81% [18]. Several studies have emphasized the effectiveness of antiviral treatment in patients with high-grade HCMV density in tissues, regardless of the cut-off value used [18,32,40]. Therefore, it was concluded that the response rates depend on the HCMV viral load in colonic tissue [17,19,44]. Regarding the treatment approach, Pillet et al. proposed a treatment strategy based on the viral load detected in the colonic mucosa. In cases of high-grade HCMV density (250 copies/mg, or more than four inclusions on IHC), both antiviral and anti-TNF alpha treatment will be initiated. In instances of low-grade HCMV density, only antiviral medication is administered in the presence of severe disease indicated by the presence of ulcers [17]. Similar to Mourad et al., they recommend antiviral treatment only in cases of severe colitis, particularly if it is unresponsive to steroids and if multiple inclusion bodies are evident, regardless of the HCMV-DNA value or when high-grade HCMV density is detected without highlighting inclusion bodies [19]. Due to the lack of specific guidelines regarding who should or should not receive treatment [44], we administered treatment in all three cases presented. The combination of ganciclovir with valganciclovir or valganciclovir alone proved effective, with no recorded relapses after one year. In the third case, the detection of CMV during histopathological examination was unexpected, and treatment was initiated due to the presence of hepatitis. However, the patient’s intestinal symptoms resolved without escalating the UC treatment. In terms of UC treatment, the continuation of therapy with Mesalazine and Adalimumab did not affect the outcomes of patients experiencing HCMV reactivation in the cases of patients 2 and 3. However, corticosteroid treatment was taped for patient 1, and Azathioprine was stopped for patient 2.

As with the majority of studies, the current article is subject to limitations. The first limitation of this research is represented by the fact that part of the conclusions is based only on the three cases presented, because it is very difficult to find a large population. Second, the review part is a short one to complement the observations from the presented cases.

There are many unknowns regarding the interaction of HCMV with the host organism. Currently, there are no established guidelines for determining whether the resolution of HCMV colitis has been achieved, nor about follow-ups or monitoring standards. One of the biggest gaps in knowledge is the latency period. In the future, if this mechanism of viral latency could be understood, perhaps it would be possible to develop a vaccine.

## 5. Conclusions

Close communication between the histopathologist and the clinician is essential. An accurate diagnosis relies on analyzing relevant clinical data in conjunction with histological findings. HCMV reactivation can occur in patients without associated risk factors. It is important to maintain a high level of clinical suspicion for CMV reactivation, especially before initiating more aggressive treatments. An increase in IgG levels may indicate recent HCMV reactivation. However, further studies are needed to validate this assumption.

## Figures and Tables

**Table 1:** Main studies, from the last 10 years, that evaluated the impact of HCMV on UC.

Studies by Chronological Order	No. of Patients Type of Study	Main Results
Nakase et al. (2014) [2]	Focused review	HCMV infection could spread from perivascular stromal cells to endothelial and epithelial cells
Kim et al. (2014) [26]	229 UC patients/83 with HCMV Original article	HCMV antigenemia assays and blood DNA PCR seem to have low sensitivities for diagnosing HCMV colitis in patients with moderate to severe UC
Skula et al. (2015) [24]	333 patients UC-HCMV colitis Clinical review article	Antiviral therapy is not required for all patients with UC-HCMV colitis
Zidar et al. (2015) [27]	10UC and 2CD/ 12 with HCMV Original article	The highest densities of HCMV-positive cells were found in the base of ulcers (qPCR 10–3809 viral copies/mg) or the edge of ulcers (qPCR 35–1049 viral copies/mg).
Yangzhen et al. (2015) [28]	Case report	HCMV colitis and UC synchronously developed
Ciccocioppo et al. (2015) [29]	24UC, 16CD, and 40 controls /30 with HCMV Original article	Antiviral therapy is more effective in refractory patients with a high viral load
Gauss et al. (2015) [30]	297 IBD patients/ 21 with HCMV Original article	HCMV reactivation in patients with risk factors is associated with a longer hospital stay (*p* < 0.001)
McCurdy et al. (2015) [31]	45UC, 21 CD, and 2 undifferentiated colitis/68 with HCMV Original article	HCMV reactivation is associated with refractory disease (OR = 3.69, *p* < 0.001) and endoscopic ulcers (OR = 2.95, *p* < 0.001)
Jones et al. (2015) [32]	1111 IBD patients/ 68 with HCMV article	Antiviral therapy improved the surgical-free survival outcome
Hirayama et al. (2016) [10]	149 UC patients/ 34 with HCMV Original article	Corticosteroid dose > 400 mg for 4 wks, extensive colitis, and a specific endoscopic finding of a punched-out ulcer are risk factors for HCMV colitis
Pillet et al. (2016) [17]	Review	Antiviral therapy and anti-TNF should be started in patients with risk factors, whatever the density of infection
Bonta et al. (2016) [20]	1023 IBD patients/12UC and 2CD with HCMV Original article	All IHC -positive biopsies for HCMV were obtained from inflamed mucosa. The average number of biopsies was eight
Lee et al. (2016) [33]	149 patients with acute severe UC (ASUC)/50 with HCMV Original article	Hospitalized patients with ASUC should be appropriately assessed for HCMV colitis.
Nolan et al. (2017) [7]	Case study	HCMV can affect immunocompetent adults, producing an illness with fever, fatigue, and myaligias
Paul et al. (2018) [34]	132 IBD patients/ 41 with HCMV Original article	Dual CMV DNA qPCR (biopsy + plasma) is a better diagnostic method than histopathology and CMV IgM serology.
Jenzer et al. (2020) [14]	Review	Antiviral therapy combined with anti-TNF should be preferred in cases of moderate or high viral loads
Mourad et al. (2020) [19]	Review	High-grade HCMV disease indicates that the virus is acting as a pathogen
Yadav et al. (2020) [35]	Review	UC, HCMV-colitis, and UC-HCMV-colitis are symptomatically and endoscopically similar
Yokoyama et al. (2020) [36]	Review	In histological negative cases, the quantitative PCR method is a promising alternative approach
Bonfanti et al. (2020) [37]	Case report	Vedolizumab therapy seems to be associated with a low side-effects profile
Park et al. (2021) [13]	Review	Anti-TNF agents may be useful for treating HCMV colitis complicating UC
Qin et al. (2021) [21]	Review	Patients with risk factors should be proactively screened for HCMV reactivation
Gilmore et al. (2022) [9]	Review	Antiviral therapy should be started in patients with steroid-refractory colitis, particularly where serum viral loads are high or multiple inclusions
Maresca et al. (2023) [6]	Review	CMV colitis is a possible determinant of a lack of a response to new biological drugs
Essen et al. (2024) [38]	81 patients with UC/51 with HCMV Article	The presence of steroid resistance associated with blood HCMV-DNA and IHC positivity increases the specificity of tissue HCMV-DNA positivity.
Momayaz Sanat et al. (2024) [23]	Review	The approach to HCMV colitis should be based on individualized assessment

## Data Availability

The original contributions presented in the study are included in the article, further inquiries can be directed to the corresponding authors.
